# Development of a Low-Cost Portable Iontophoresis Device

**DOI:** 10.7759/cureus.98797

**Published:** 2025-12-09

**Authors:** Drin Rrmoku

**Affiliations:** 1 Electrical Engineering/Biomedical Engineering, Independent Electrical Engineer, Prishtina, ALB

**Keywords:** direct current therapy, home treatment, hyperhidrosis, iontophoresis device, low-cost device, medical electronics, palmar hyperhidrosis, portable medical device, sweat gland inhibition, tap water iontophoresis

## Abstract

Hyperhidrosis, particularly of the palms and soles, is a common condition characterized by excessive and uncontrollable sweating that can significantly affect daily activities and quality of life. Among the many various treatments, the iontophoresis has emerged as a safe, simple, and non-pharmacological solution.

Iontophoresis is used to treat excessive sweating (hyperhidrosis) by temporarily disabling sweat glands through a small direct current through the skin via water-filled electrodes. However, such devices are not widely available, and the price can range from hundreds to thousands of dollars.

This study presents the design and working principle of a portable, low-cost iontophoresis device with a compact form factor suitable for home use. The system is powered by a standard USB power bank, providing long operating time, easy recharging, and improved portability compared to conventional mains-powered units.

The prototype iontophoresis device is capable of delivering a controlled current of up to 25 mA. The device remained fully portable during use and operated reliably when powered from a standard USB power bank. As a functional observation of the system’s current delivery, a noticeable difference in sweat reduction was observed between the two treated hands. Only the hand connected to the anode during all three sessions showed a marked decrease in sweating, while the other hand showed no measurable change.

Commercial iontophoresis systems are often priced in the hundreds or even thousands of dollars despite being based on low-power electronic circuitry and readily available materials. Their high cost is influenced by factors such as regulatory approval requirements, safety testing, and limited market competition. The results of this work demonstrate that a functional, portable, and safe iontophoresis device can be produced at a fraction of this cost using basic components.

## Introduction

Hyperhidrosis is a condition where the sweat glands become overactive and cause sweating far beyond what is needed for normal temperature regulation [1]. The armpits, palms, soles, and face have the highest number of eccrine sweat glands, making them the areas most commonly affected by hyperhidrosis [2].

Research suggests that hyperhidrosis affects approximately 3% of the US population, making it a relatively common condition. Because the sweating is excessive and often unpredictable, many individuals experience significant emotional and psychological distress. The condition may also interfere with daily tasks, such as writing, gripping objects, or using electronic devices, and can create challenges in professional settings where frequent hand use or close interaction with others is required. As a result, hyperhidrosis can impact overall quality of life far more than its physical symptoms might suggest [3].

Among the many various treatments, the iontophoresis has emerged as a promising solution [4], which is considered to be safe, simple, and is a non-pharmacological treatment that works without the use of any drugs or chemical agents.

Iontophoresis involves passing a low direct electric current through two electrodes through an electrolyte solution [5]. The use of electrolyte solution or moist pads between the electrode plate and skin is necessary for making a perfect contact, preventing any skin burns, overcoming skin resistance, and protecting the skin from absorbing any caustic metallic compound formed on the metal plate surface [6].

Currently, it is not known why iontophoresis halts or diminishes sweating, but it is believed that iontophoresis may cause a functional impairment of the sweat gland, either by completely blocking sympathetic nervous system transmission to the gland, raising the threshold for transmission of sympathetic nerve impulses, or changing the cellular secretory physiology [7].

Treatment is typically performed for about 20 minutes every two to three days [8] or for 10 minutes three to five times per week [7].

Commercial iontophoresis devices are often priced in the several-hundred-dollar range [9], which places them beyond the reach of many users who require frequent or long-term treatment. Their high cost is largely driven by proprietary designs, specialized components, and limited market competition. As a result, affordability becomes a significant barrier, especially in settings where patients seek simple home-based therapy options.

The purpose of this paper is to describe how a simple and portable iontophoresis device can be built at low cost. Although the effectiveness of the treatment is not the focus of this work, a small functional observation from trying the prototype is included to demonstrate basic operation.

## Technical report

Design approach

The proposed iontophoresis device was developed using readily available components and simple modules, with a strong focus on keeping the design low-cost and highly portable.

**Figure 1:**
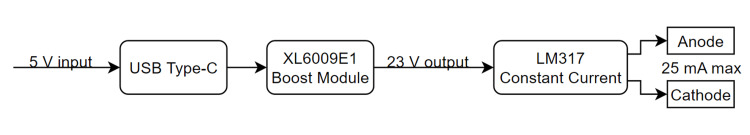
1 Block diagram of the iontophoresis device Image Credit: Designed by the author

The overall circuit design is shown in Figure 1. The device is powered through a USB Type-C connector using a standard 5 V power bank. The voltage is then increased to approximately 23 V using an XL6009E1 boost converter module to provide enough headroom for stable current regulation. The boosted voltage feeds an LM317-based constant current circuit, which limits the output to a maximum of 25 mA. The regulated current is delivered to the anode and cathode electrodes placed in the water trays.

**Figure 2:**
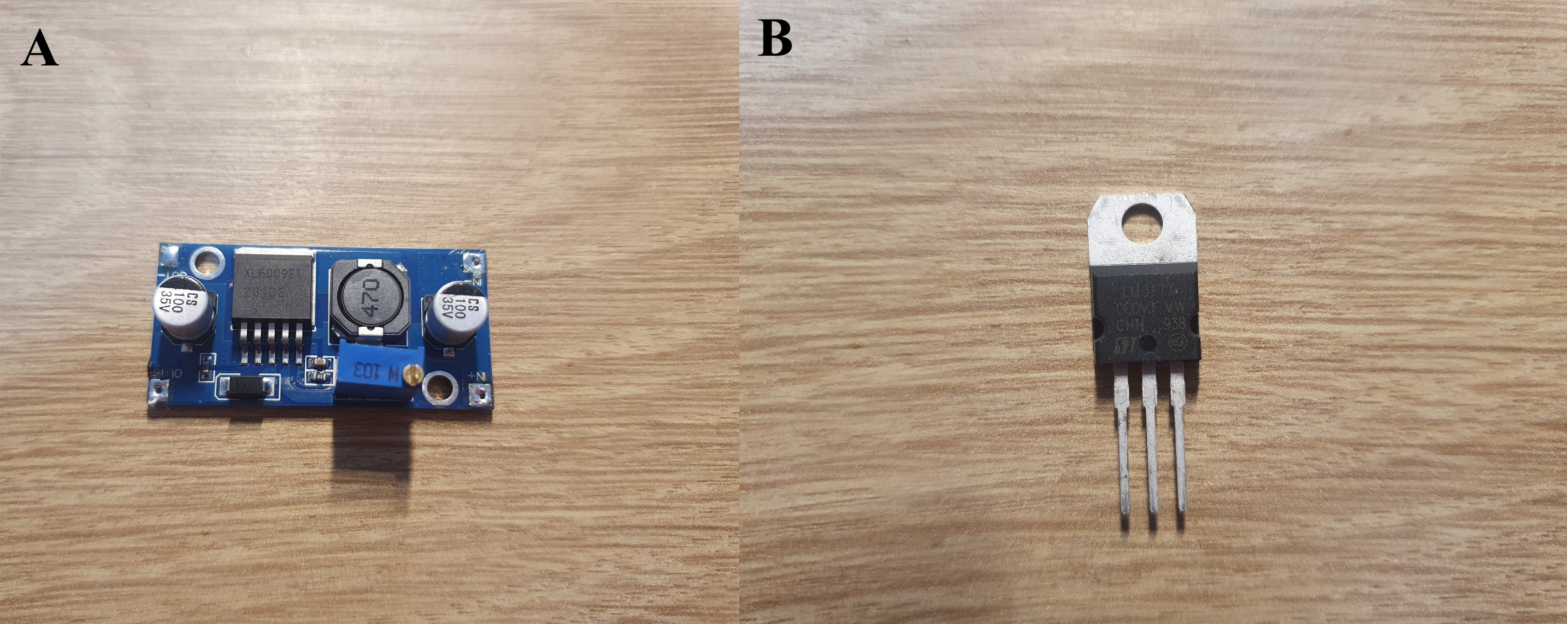
Components used in the iontophoresis prototype A: XL6009E1 DC to DC boost converter module is used to raise the 5 V input to approximately 23 V. B: The LM317 voltage regulator is configured as a constant current source for driving the electrodes.

Figure 2 shows the two main components used in the construction of the iontophoresis device. The XL6009E1 boost converter module, shown in panel A, increases the 5 V supplied by a power bank to a higher voltage needed for stable current regulation. Panel B displays the LM317 regulator, which is configured as a constant current driver to limit the output to safe levels during treatment.

**Figure 3:**
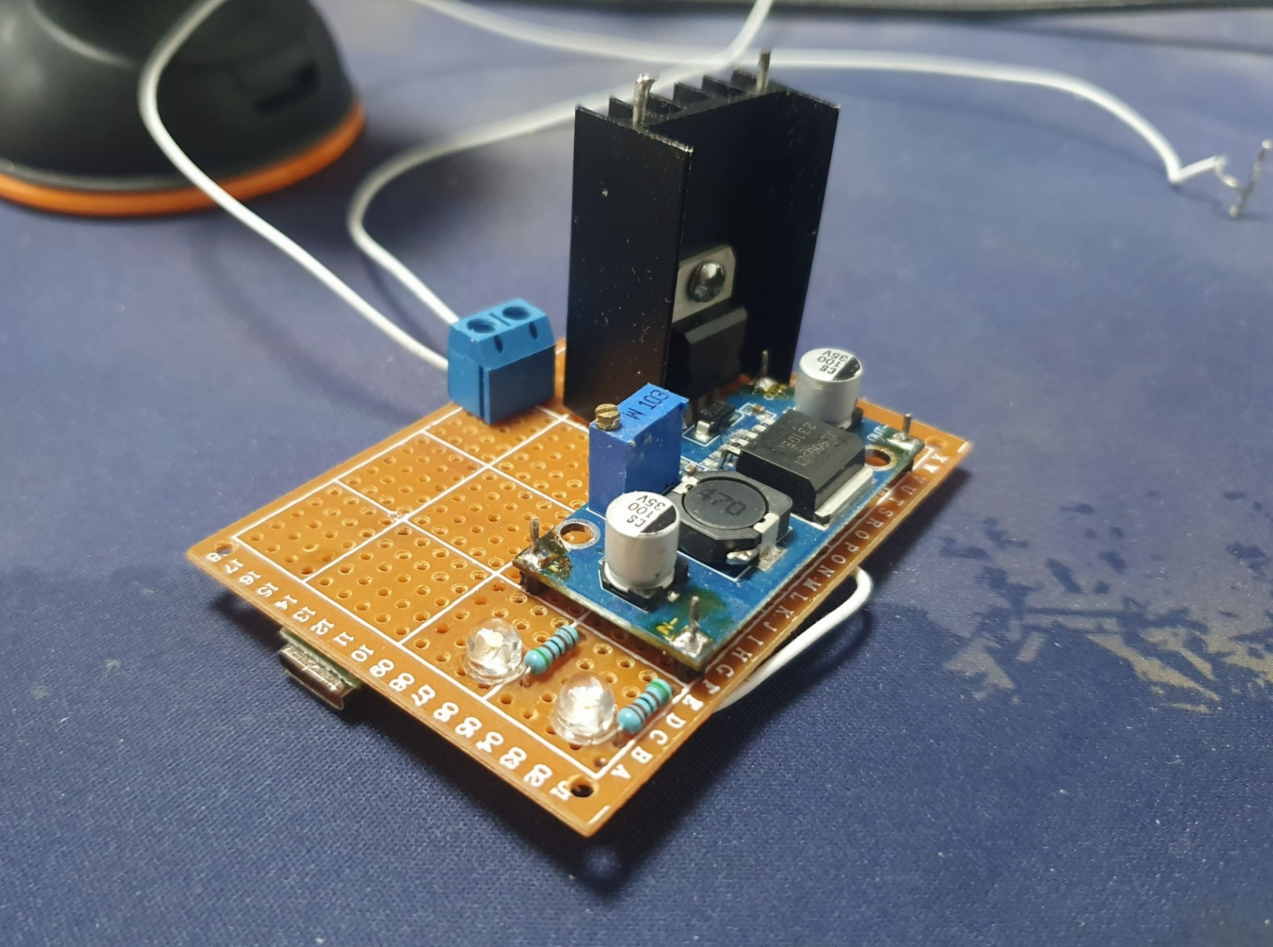
Prototype of the iontophoresis device

Figure 3 shows the assembled prototype with a heatsink on the LM317 to avoid overheating, along with a screw terminal for easy connection of the anode and cathode.

Results

**Figure 4:**
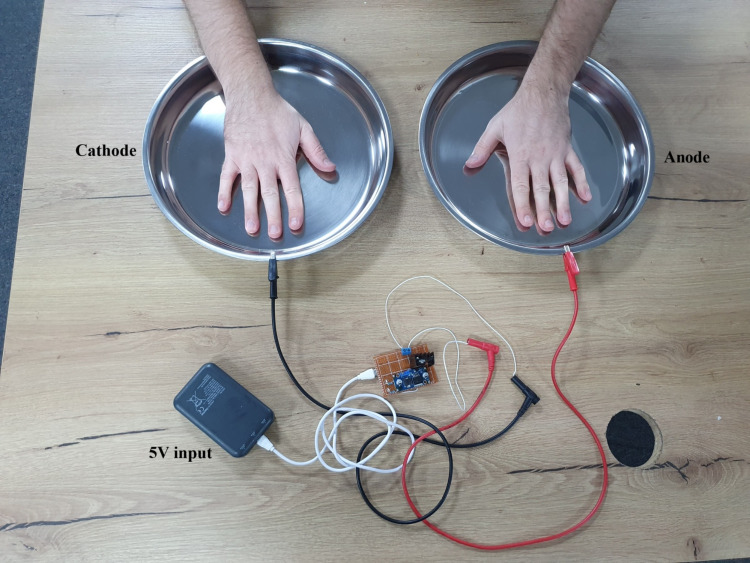
Setup of the iontophoresis prototype during hand immersion testing The prototype device is shown connected to two stainless steel trays serving as the cathode and anode, respectively. A 5-V power source supplies the circuit, and the hands are placed in separate trays to complete the iontophoresis pathway.

As shown in Figure 4, two stainless steel trays were used as the anode and cathode. Using non-corrosive materials is important to prevent corrosion and metal particles from entering the water, which can lead to skin irritation.

Both trays are filled with tap water at a temperature that one can comfortably hold their hand on.

Because this device was developed as a low-cost do-it-yourself (DIY) prototype, it was not designed to meet formal medical device standards, and the early current control relied only on a simple resistor network and the limits of the power supply. This resulted in the current approaching its maximum value of approximately 25 mA during the first tests, which explains the mild tingling sensation and the brief shock-like feeling when placing the hands into or out of the water. Subsequent revisions incorporated a dedicated current-limiting stage and improved insulation during hand placement, which stabilized the current and eliminated these sensations.

Three treatment sessions per week were performed using this setup for around 10 minutes each. During all sessions, the anode was consistently placed on the left hand and the cathode on the right hand to observe any differences in effect between the two electrode polarities.

Approximately five to six days after the third session, a noticeable change was observed.

**Figure 5:**
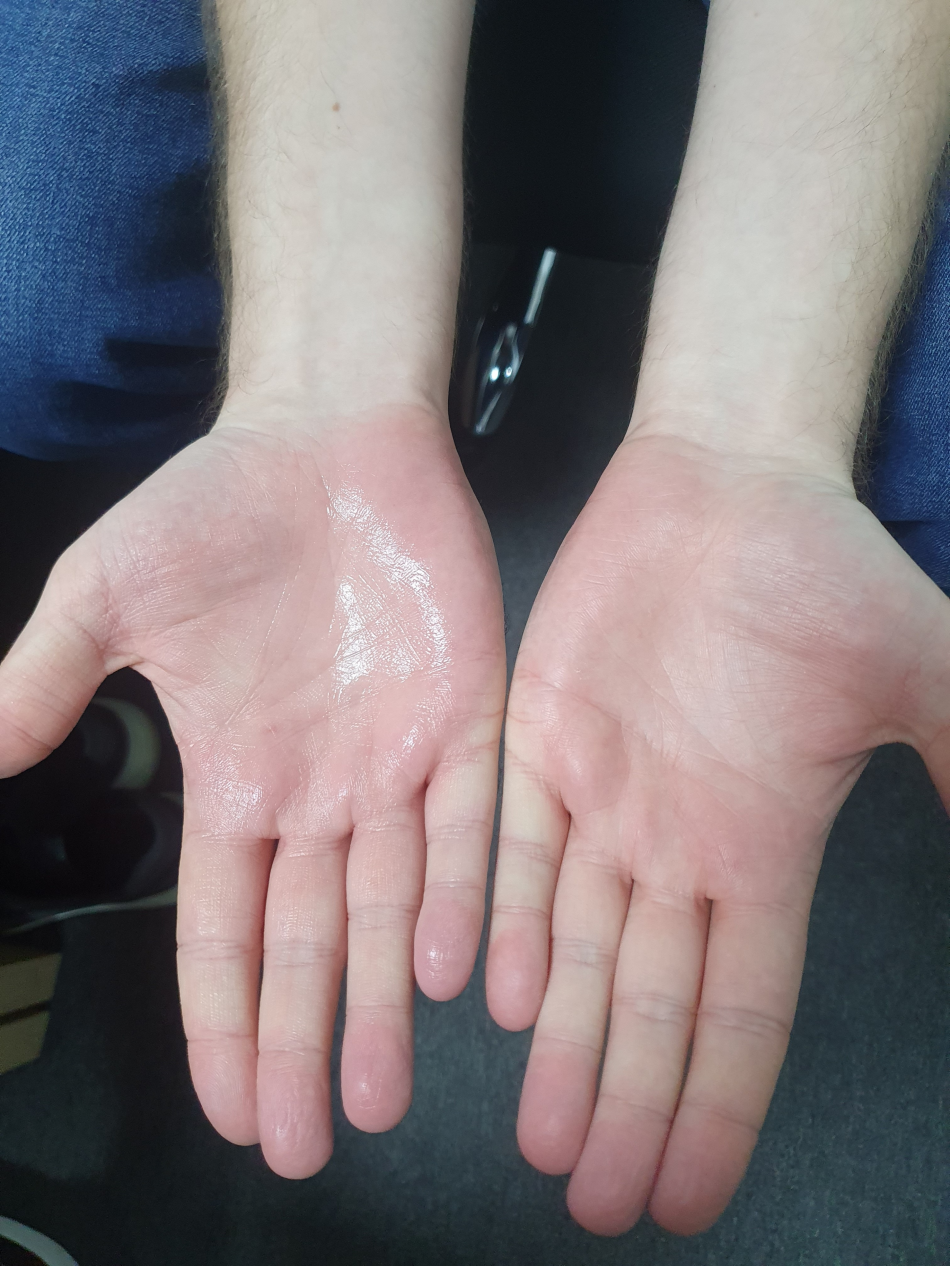
Comparison of palmar moisture after three iontophoresis sessions The left hand, which was connected to the anode during all treatments, shows visible residual moisture, while the right hand, connected to the cathode, appears noticeably dry. This illustrates the difference in sweating between the two electrode positions.

As seen in Figure 5, there was a noticeable reduction of moisture on the left hand, which had been connected to the anode during all three sessions, while the right hand, connected to the cathode, did not show any significant change.

It was also noted that after a week, the overall condition of the skin on the left hand showed visible improvement.

**Figure 6:**
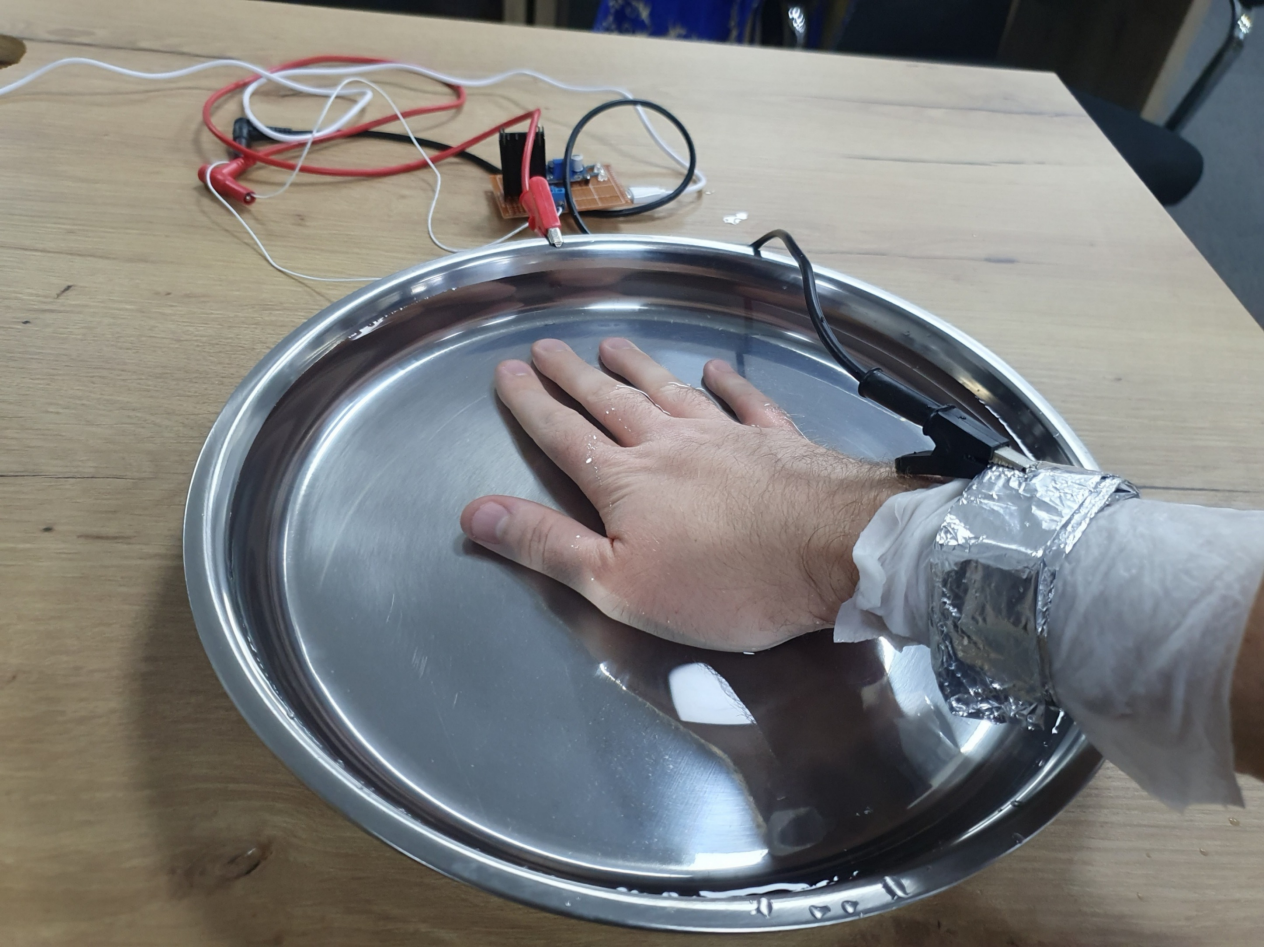
Modified current path using an insulated wrist barrier during iontophoresis This modification was implemented to avoid a hand-to-hand current pathway, reducing the risk of electrical flow across the chest and ensuring that the current remains localized to the treated hand.

During the development of the prototype, a specific safety concern was considered: hand-to-hand current flow can create a pathway across the chest, allowing electrical current to pass through the heart. This configuration is generally avoided in electrotherapy and biomedical device design because current traveling through the thoracic region may interfere with normal cardiac conduction. To avoid any possibility of hand-to-hand current flow through the thoracic region, the current delivery method was modified by placing the return electrode on the same arm being treated, as shown in Figure 6, insulated from the skin with a moist towel to prevent direct metal contact. This configuration kept the current confined to a single limb and prevented it from traveling across the chest.

To avoid the current pathway and the risk of electrical flow across the chest, the cathode was placed on the wrist and isolated from the skin with a wet towel. The duration of the treatment’s effect is still uncertain and requires further observation.

## Discussion

Hyperhidrosis is a condition that affects thousands of people around the world. It causes excessive sweating far beyond what the body needs for thermoregulation [1]. Because the armpits, palms, soles, and face contain high densities of eccrine sweat glands, these areas are most commonly affected [2]. This can interfere with routine daily tasks, and many individuals also experience emotional and psychological distress.

Among the available treatment options, iontophoresis has been presented as a promising temporary solution [4]. The technique works by passing a low level of direct electrical current between an anode and a cathode through an electrolyte solution, which in many cases is simply tap water [5], with the resulting current density influenced by water conductivity, electrode placement, and the natural resistance of the skin. Although the exact mechanism is not fully understood, iontophoresis is believed to decrease sweating by impairing sweat gland function, possibly by blocking sympathetic nerve transmission, increasing the threshold for nerve activation, or altering the cellular physiology involved in sweat secretion.

Commercial iontophoresis devices are often priced in the several-hundred-dollar range [9], which makes treatment difficult to access for individuals who require frequent or home-based sessions. The aim of this paper is to describe the design and function of a small, portable iontophoresis device that can be used for home treatment. Although demonstrating treatment effectiveness is not the primary focus of this work, a functional observation from testing the prototype is included.

Traditional commercial iontophoresis units typically deliver current in the range of 10 to 20 mA and rely on mains-powered systems [7,8]. Compared with these devices, the prototype in this study delivers a controlled current of up to 25 mA, which is consistent with current levels described as effective in earlier literature [7]. In addition, prior research has noted that consistent polarity use, especially prolonged anodal exposure, may influence treatment outcomes [7], which aligns with the observed reduction in sweating on the anode-treated hand in this work. Furthermore, commercial devices remain costly despite using simple circuitry; a concern is also highlighted in recent dermatology literature that discusses affordability barriers in iontophoresis treatment [9]. By using readily available components and a USB power source, the device presented here offers a lower-cost and more portable alternative compared to existing systems while still demonstrating functional current delivery.

Safety and limitations

This project describes a DIY iontophoresis device assembled from basic electronic components. Because the system delivers electrical current through water, improper construction or use may expose individuals to risks such as electrical shock, skin irritation, burns, or unintended current pathways. The design presented here has not undergone formal safety testing, regulatory evaluation, or clinical validation, and is intended solely for educational and proof-of-concept purposes. Anyone attempting to replicate or use a similar device should do so with caution and ideally under the supervision of a qualified professional. Further engineering refinement, safety assessment, and controlled testing would be required before such a device could be considered suitable for home or clinical use.

## Conclusions

This work demonstrates that a simple, low-cost, and fully portable iontophoresis device can be constructed using readily available electronic components while still delivering a stable current suitable for treatment. The prototype operated reliably from a standard USB power bank and produced a clear functional difference between the anode-treated and cathode-treated hands, indicating that the system is capable of performing basic iontophoresis. Although the short-term observation suggests a reduction in sweating on the treated side, the duration and consistency of this effect remain uncertain and require further evaluation. Overall, the results suggest that accessible and affordable iontophoresis devices are technically feasible, offering a potential low-cost alternative for individuals who cannot access commercial systems.
